# Ada-LT IP: Functional Discriminant Analysis of Feature Extraction for Adaptive Long-Term Wi-Fi Indoor Localization in Evolving Environments

**DOI:** 10.3390/s24175665

**Published:** 2024-08-30

**Authors:** Tesfay Gidey Hailu, Xiansheng Guo, Haonan Si, Lin Li, Yukun Zhang

**Affiliations:** 1 Department of Software Engineering, Addis Ababa Science and Technology University, Addis Ababa 16417, Ethiopia; tesfaygidey21@std.uestc.edu.cn; 2 Department of Information and Communication Engineering, University of Electronic Science and Technology of China, Chengdu 611731, China; sihaonan@std.uestc.edu.cn (H.S.); 201811011926@std.uestc.edu.cn (L.L.); yukunzhang@std.uestc.edu.cn (Y.Z.)

**Keywords:** indoor localization, Wi-Fi fingerprinting, functional discriminant analysis, transfer learning, features extraction, computational complexity

## Abstract

Wi-Fi fingerprint-based indoor localization methods are effective in static environments but encounter challenges in dynamic, real-world scenarios due to evolving fingerprint patterns and feature spaces. This study investigates the temporal variations in signal strength over a 25-month period to enhance adaptive long-term Wi-Fi localization. Key aspects explored include the significance of signal features, the effects of sampling fluctuations, and overall accuracy measured by mean absolute error. Techniques such as mean-based feature selection, principal component analysis (PCA), and functional discriminant analysis (FDA) were employed to analyze signal features. The proposed algorithm, Ada-LT IP, which incorporates data reduction and transfer learning, shows improved accuracy compared to state-of-the-art methods evaluated in the study. Additionally, the study addresses multicollinearity through PCA and covariance analysis, revealing a reduction in computational complexity and enhanced accuracy for the proposed method, thereby providing valuable insights for improving adaptive long-term Wi-Fi indoor localization systems.

## 1. Introduction

**Figure 1:**
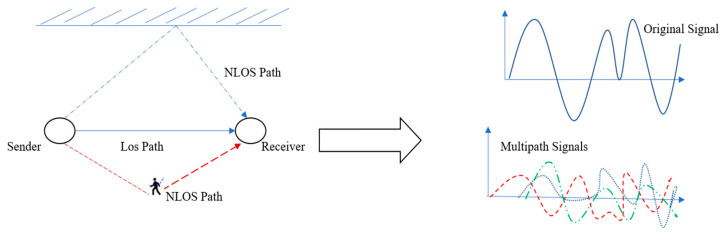
Multipath received signal effect of indoor environment scenario.

With the advent of the Internet-of-Things (IoT), along with the rollout of 5G and emerging 6G technologies, the significance of location-based services (LBS) has markedly increased. Accurate indoor positioning information is essential for a range of applications, including business location services, data mining, security monitoring, and venue management [1,2,3,4]. While global positioning system (GPS) technology operates effectively in outdoor settings, it proves inadequate for indoor localization due to weak signal reception in complex environments. Key challenges include limited line of sight, insufficient satellite signal penetration, and interference from internal obstacles, such as shadows and multipath fading [5,6,7,8,9]. As urbanization intensifies and a majority of activities shift indoors, the demand for reliable indoor positioning systems (IPSs) has surged. A variety of wireless technologies have emerged to address this need, including radio frequency identification (RFID) [10], Bluetooth [11], ultra-wideband (UWB) [12], Zigbee [13], inertial navigation [14], and visible light communication (VLC) [15]. However, the implementation of these technologies often incurs significant infrastructure costs. Effective IPSs leverage diverse signal characteristics—such as received signal strength (RSS), channel state information (CSI), angle of arrival (AOA), and time of arrival (TOA)—to accurately locate objects or individuals in environments where GPS signals are compromised. To meet the demands of indoor settings, these systems must provide high accuracy, rapid estimation times, and low power consumption. Nevertheless, the dynamic nature of indoor environments introduces variability in signal patterns, which can adversely affect positioning performance [16,17,18]. To achieve a balance between computational costs and accuracy, IPSs must optimize available resources while accounting for environmental factors and maintaining an acceptable margin of error. The mission of the application and the overall system cost are also critical determinants of positioning performance [19,20,21]. Among the various indoor positioning technologies, Wi-Fi fingerprint-based IPS (FPBIPS) stands out as a particularly promising solution owing to its cost-effectiveness and ease of implementation. However, FPBIPS is susceptible to challenges posed by multipath effects, shadowing, and scattering, which are influenced by the dynamic nature of indoor environments [22,23,24]. Additionally, signal attenuation in wireless communication systems—primarily attributed to path loss, shadowing, and multipath effects—can significantly degrade location accuracy [25]. Figure 1 illustrates the impact of multipath on the received signal within an indoor setting.

The variability of fingerprint values in indoor environments, influenced by factors such as device heterogeneity, measurement timing, user orientation, and channel conditions, significantly impacts positioning performance. This dynamic variability often leads to mismatches between stored and real-time fingerprints, posing a critical challenge for accurate indoor positioning. To address these issues, various fingerprint-matching strategies have been developed [26,27,28], broadly categorized into deterministic [29,30,31] and stochastic approaches [32,33,34]. To mitigate the challenges posed by complex indoor signal fluctuations, several FPBIPS methods have been proposed. One approach involves modeling signal jitter using the path loss model; however, this method is constrained by its dependence on map information and the assumption of a fixed receiver position [35,36,37]. In addition, machine learning (ML) algorithms have also been applied to RSS fingerprint-based indoor positioning problems, yet these techniques often fail to consider critical factors, such as leveraging related source domains, which could enhance the overall positioning accuracy and reduce the labor-intensive costs associated with offline fingerprint data collection [38,39,40]. In addition to that, recent advancements in addressing the inherent challenges associated with FPBIPS have been extensively documented in the literature. Various studies have proposed innovative algorithms and methodologies aimed at enhancing the resilience of these systems against signal fluctuations and the deterioration of fingerprints over time due to the dynamic nature of indoor environments [41,42,43,44,45]. For instance, advanced techniques and machine learning approaches have been demonstrated to significantly improve accuracy and robustness in environments with fluctuating signals and evolving conditions [44,45]. A novel multi-modal indoor localization method that integrates visual information, Wi-Fi signals, and lidar data, achieving high precision with an average 3D localization accuracy of 0.62 m and a mean square error of 1.24 m in two-dimensional tracking [44]. The study highlights the potential of hybrid techniques in enhancing location-based services within complex environments. Nevertheless, the performance relies on the accuracy and compatibility of the multimodal sensors used. In addition, the joint processing of multiple data sources might introduce additional overhead costs, which could limit deployment on low-power devices.

Furthermore, achieving desired accuracy with RSS-based fingerprinting requires a large number of labeled samples, which is expensive and time-consuming. Crowdsourcing approaches have been studied to create and update radio maps, aiming to eliminate the need for site surveying [46,47,48]. Algorithms are being developed to generate radio maps using user traces collected from the crowd. However, trace-matching algorithms based on inertial sensors often face issues with unstable posture and high-power consumption of smartphones [49,50,51]. While our work focuses on single-signal metrics, hybrid methods combining Bluetooth, Wi-Fi, UWB, and ZigBee [52] have been proposed to enhance indoor positioning. Other examples include the integration of Wi-Fi with Visual Light Positioning (VLP) [53] and Bluetooth Low Energy (BLE) [54]. A novel localization framework has been developed that integrates GNSS, Wi-Fi Fine Time Measurement (FTM), and built-in sensors to achieve precise meter-level accuracy [41]. The framework utilizes advanced techniques, including pedestrian dead reckoning and an adaptive multi-model extended Kalman filter, to ensure seamless indoor and outdoor positioning. Experimental results demonstrate substantial improvements in localization reliability, making it highly suitable for complex environments [41]. However, the framework’s reliance on multiple data sources and algorithms can introduce complexity, requiring significant computational resources and careful calibration. Moreover, although these hybrid approaches can achieve meter-level localization accuracy, they may introduce complexities in system integration and increase overall costs. These contributions underscore the ongoing efforts to refine IPS performance in complex indoor settings while acknowledging the inherent limitations. In addition, a recent study in [55] has also proposed an innovative indoor localization system named iToLoc, which combines adversarial learning and semi-supervised techniques to address the limitations of existing FPBIPS methods. By utilizing a domain adversarial neural network, iToLoc effectively mitigates issues related to signal variability and device differences, achieving a localization accuracy of 1.92 m with over 90% success rate even after several months of operation. However, the impact of signal sampling fluctuations, the application of various data reduction techniques to extract significant predictors, and the use of positive knowledge transfer, which are critical aspects, have been overlooked in addressing the major challenges of indoor localization. Thus, in this paper, we propose a functional discriminant analysis method for feature extraction in Wi-Fi indoor localization systems. This paper employs advanced data reduction techniques to mitigate the overhead of fingerprint calibration by transforming Wi-Fi RSS values into a novel vector using linear transformation. The goal of this research paper is to enhance indoor localization performance for adaptive long-term Wi-Fi indoor positioning (adaptive LT Wi-Fi IP) by maximizing variance in a lower dimension while reducing computational complexity.

**Figure 2:**
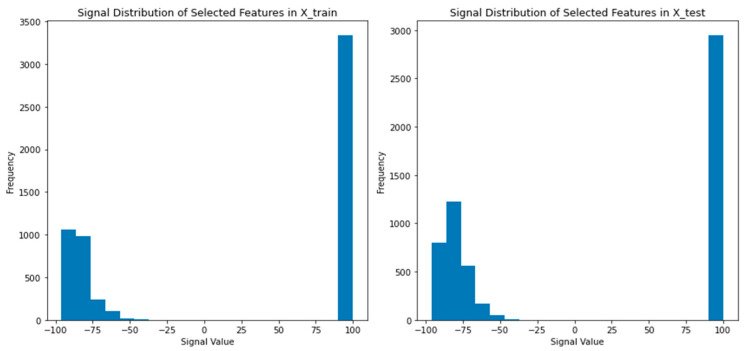
Signal distribution comparison between training and testing sample.

This study examines the temporal fluctuations in signal strength and proposes the implementation of transfer learning methodologies to enhance model performance in indoor positioning, even in scenarios with limited training data [56]. However, a key limitation of this approach lies in the presumption of similar data distributions between the training and testing datasets; discrepancies in these distributions can significantly impact model accuracy and reliability [57]. The dynamic nature of indoor environments is underscored by substantial variations in signal distributions observed between the training and testing datasets, as confirmed by the Mann–Whitney U test (see Figure 2). To mitigate this challenge, the study highlights the necessity for developing adaptable models capable of accommodating these environmental variations. Thus, the contributions of this paper include:

(1) We propose the application of functional discriminant analysis (FDA) in combination with transfer learning techniques to tackle the challenge of high offline fingerprint calibration overhead. To achieve this, we generate new feature spaces that focus on the most significant predictors. These predictors enhance the separability of the model, leading to improved accuracy in indoor positioning estimates.
(2) We examined the impact of sampling signal fluctuations on different algorithms in indoor localization scenarios. Multiple training samples were used to assess the influence of sampling fluctuations, while all collected testing samples for each month were used to evaluate algorithm robustness.
(3) We applied covariance analysis (CA) to reduce the multicollinearity problem of the various RSS values collected at a reference point (RP), aiming to minimize computational complexity.
(4) We compare the performance of different feature extraction methods, namely mean signal values, principal component analysis (PCA), and linear discriminant analysis (LDA/FDA), for adaptive LT Wi-Fi IP. We evaluate the effectiveness of these methods based on the achieved metrics and also investigate the hybrid effect of combining features extracted from multiple methods.

The rest of the paper is organized as follows: Related works are presented in Section 2. Section 3 describes fingerprinting localization framework and its problem formulation. Experimental results, discussions, and evaluation metrics are presented in Section 4. Conclusions and recommendations are provided in Section 5. 

## 2. Related Works

This study presents an overview of IPSs and explores the application of FDA for feature extraction in this domain. Indoor positioning (IP) has become an increasingly important research area, with applications in smart buildings, emergency response, and location-based services [58,59,60]. The paper discusses two main approaches for Wi-Fi-RSS-based IPSs: path loss model-based and fingerprinting. The path loss model-based approach relies on the relationship between RSS and distance to determine the target object’s location [61,62,63]. However, due to the complex indoor environment, including factors such as non-line-of-sight (NLOS) propagation, multipath effects, and a dynamic environment, this distance-based approach cannot provide accurate geometric parameters [64,65]. In contrast, the fingerprint-based approach has gained significant attention in indoor localization as it does not rely on estimating geometric parameters and performs better than the distance-based approach in complex indoor environments [66,67]. Not only that, but also Wi-Fi-based RSS fingerprinting has gained popularity due to its advantages, including universal availability, privacy protection, and low implementation cost [22,23,24]. It is extensively used for communication purposes and holds great importance for terminals and sensor networks in IoT applications [1,2,3,4]. This approach involves two main phases: first, RSS fingerprints are collected from each Wi-Fi access point (AP) at multiple locations to create a radio map or fingerprint database, and then a predictive model is trained to establish the relationship between the signal and location [35,36,37]. 

However, this method has faced criticism for the high cost of creating wireless maps, which can be very expensive [44,45,46]. Attempts have been made to reduce the effort and time required for radio map generation, such as crowdsourcing [45] and simultaneous Wi-Fi localization and mapping, but these approaches have their own limitations [68]. Moreover, existing Wi-Fi networks are primarily designed for communication rather than positioning, and there is a need for robust and efficient algorithms to enhance their positioning performance. Nevertheless, the fingerprint-based approach still faces challenges in achieving robust and efficient positioning performance in dynamic indoor environments. Researchers have proposed several methods to deal with the dynamic indoor environment, which leads to low localization accuracy due to variations in fingerprint patterns over time. These methods can be classified into four groups: (i) probabilistic methods [69,70], (ii) machine learning methods [71,72], (iii) exploiting the quality of fingerprints of various signal features [73,74,75,76], and (iv) a fused group of fingerprints [77,78]. Although these methods have improved the location accuracy, they still suffer from fluctuations in the signal distribution and are not robust in indoor dynamic environments. Hybrid location systems (HPS) have been proposed to solve the single location problem, and the results demonstrate better location performance than the single system [79,80]. However, a hybrid base station falls outside the scope of this work and is not economically feasible. Additionally, computational complexity is a serious problem for hybrid systems based on indoor positioning. 

To address the computational complexity of IPSs, various studies have used the application of PCA for data preprocessing aimed at reducing the dimension and noises of the original dataset [81,82,83]. These methods require intensive training dataset calibration overhead. However, the distribution of signal measurements for both training and testing did not account for the long-term effects of signal variations in the complex indoor environment. Moreover, studies have proposed LDA-based algorithms to eliminate the interference of environment and noise, generating a more stable and distinguishable fingerprint [84,85,86]. Additionally, several indoor localization algorithms have been proposed in the literature to improve indoor location estimation based on the functional discriminant analysis [87,88,89]. However, these methods have not considered the critical issues of offline fingerprint calibration overhead and have utilized CSI fingerprints, which demand extra hardware infrastructure cost compared to RSS fingerprints. Thus, the primary goal of this research study is to enhance the performance of long-term adaptive indoor localization systems that utilize RSS fingerprinting by reducing the computational complexity and resource requirements, both in terms of cost and time, through the application of transfer learning techniques in combination with several data reduction methods.

## 3. Problem Formulation and Framework

We consider that the general fingerprint-based positioning of the indoor environment scenario (FPBIP) consisting of L reference points (RPs), each reference point is indexed with a label $k,\left(k=0,1,\ldots ,L-1\right)$, and p detectable Wi-Fi access points (m) were available. The $i^{th}$ Wi-Fi signal strength of fingerprints received at the $k^{th}$ reference point of the $m^{th}$ Wi-Fi AP is a vector denoted as $r_{im}^{\left(k\right)}$ and the fingerprint database can be represented as a matrix, as follows:

$$R_{im}^{\left(k\right)}=\left\{R_{im}^{\left(k\right)},\left(x_{l},y_{l}\right)\right\}={\left[r_{im}^{\left(0\right)},r_{im}^{\left(1\right)},\ldots ,r_{im}^{\left(L-1\right)}\right]}^{T};\;i=1,2,\ldots ,n_{k}\;\&\;m=1,2,\ldots ,p$$

and $\left(x_{l},y_{l}\right)$ is the corresponding coordinate to the associated location of the fingerprint. The target instances would be received during the testing phase, denoted as $R_{im}^{\left(k\right)};\;i=1,2,\ldots ,n_{t}\;\&\;m=1,2,\ldots ,p_{t}$, and the offline source domain can be represented as follows: $R_{im}^{\left(k\right)}=\left\{R_{im}^{\left(k\right)},\left(x_{l},y_{l}\right)\right\};\;i=1,2,\ldots ,n_{s}\;\&\;m=1,2,\ldots ,p_{s}$ called the labeled source data. $n_{t}$ and $n_{s}$ represent the numbers of measurements for the target and source data instances, respectively. Thus, the offline fingerprint database can explicitly be represented as follows:

(1) $$R_{im}^{\left(k\right)}=\left[\begin{array}{c} \begin{array}{ccc} r_{11}^{\left(0\right)} & \cdots & r_{1p}^{\left(0\right)}\\ \vdots & \ddots & \vdots \\ r_{n1}^{\left(0\right)} & \cdots & r_{np}^{\left(0\right)} \end{array}\\ \begin{array}{ccc} r_{11}^{\left(1\right)} & \cdots & r_{1p}^{\left(1\right)}\\ \vdots & \ddots & \vdots \\ r_{n1}^{\left(1\right)} & \cdots & r_{np}^{\left(1\right)} \end{array}\\ \begin{array}{ccc} r_{11}^{\left(k-1\right)} & \cdots & r_{1p}^{\left(k-1\right)}\\ \vdots & \ddots & \vdots \\ r_{n1}^{\left(k-1\right)} & \cdots & r_{np}^{\left(k-1\right)} \end{array} \end{array}\right]$$

**Figure 3:**
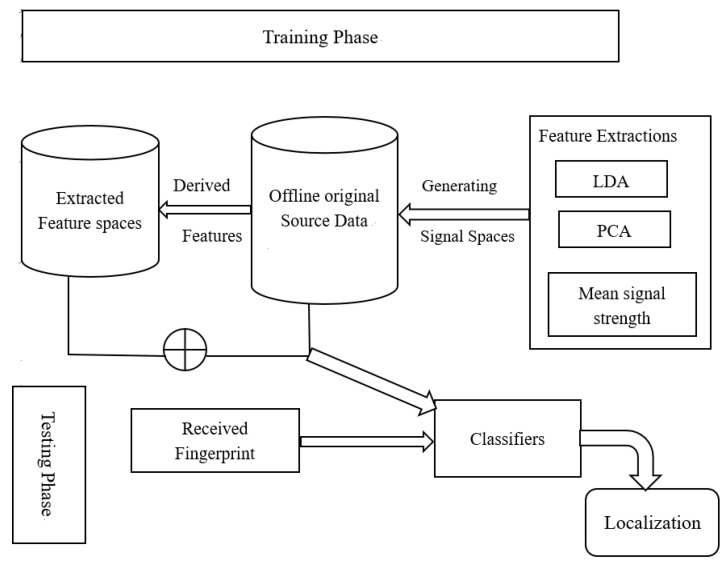
Proposed framework for Ada-LT Wi-Fi RSS fingerprint indoor localization.

The research paper introduces FPBIP, a method for creating a radio map used for online positioning. The offline phase involves generating reference points by capturing Wi-Fi APs data or wireless indoor positioning metrics along with their real-world locations during training. In the testing phase, the mobile user collects received signal strength values and sends them to a server, which uses pattern-matching algorithms to compare the measured RSS with the fingerprints in the database and determine the location. However, this fingerprint-based approach has several drawbacks. It is labor-intensive and expensive, and the radio map quickly becomes outdated due to the dynamic indoor environment. Factors such as device heterogeneity, measurement times, user and antenna orientation, and channel conditions can significantly affect positioning accuracy, leading to discrepancies between online and stored fingerprints. The limitations stem from challenges in maintaining an up-to-date fingerprint database and the high cost of using a large number of labeled samples. To address these limitations, researchers proposed hybrid location systems as an alternative, which have shown better positioning performance compared to single-system approaches. However, the computational complexity of hybrid systems poses challenges for indoor localization, and the economic feasibility of hybrid base stations is beyond the scope of this research. Figure 3 illustrates the proposed framework for adaptive long-term based on Wi-Fi RSS fingerprint indoor localization, which exploits both the knowledge from the source domains based on the mean signal received strength and target domains of heterogeneous feature spaces. The offline fingerprints of the radio map and the testing fingerprints obtained during the online phase are considered the source and target domains, respectively. In an environment that is slightly dynamic, the RSS measurements collected at a reference point $X_{k}$ are assumed to be normally distributed with mean $\mu_{k}$ and variance $\sigma_{k}^{2}$ that is $X_{k}\sim N\left(\mu_{k},\sigma_{k}^{2}\right)$. Similarly, the overall measurements collected from the available Wi-Fi APs are supposed to follow a multivariate normal distribution with the overall mean μ and multidimensional covariance ∑, such that $R_{im}^{\left(k\right)}=X\sim N\left(\mu ,\sum \right)$ as follows:

(2) $$\sum =\left(\begin{array}{c} \sigma_{1}^{2}\\ \rho_{21}\sigma_{2}\sigma_{1}\\ \vdots \\ \rho_{p1}\sigma_{p}\sigma_{1}\; \end{array}\begin{array}{c} \rho_{12}\sigma_{1}\sigma_{2}\\ \sigma_{2}^{2}\\ \vdots \\ \rho_{p2}\sigma_{p}\sigma_{2} \end{array}\begin{array}{c} \cdots \\ \cdots \\ \ddots \\ \cdots \end{array}\begin{array}{c} \rho_{1p}\sigma_{1}\sigma_{p}\\ \rho_{2p}\sigma_{2}\sigma_{p}\\ \vdots \\ \sigma_{p}^{2} \end{array}\right)$$

It is noted that each feature of the RSS measurements collected at a reference point $X_{k}$ are assumed to be independent and identically distributed such that the joint probability density function with p dimension of features of the RSS values collected certainly from the defined region of L reference points can be given as follows:

(3) $$f\left(R_{im}^{\left(k\right)},\mu \right)=\prod_{i=1}^{n}\left\{\frac{1}{{\left(2\pi \right)}^{p/2}{\left|\sum \right|}^{1/2}}e^{\frac{-1}{2}{\left(X-\mu \right)}^{\prime }\sum^{-1}\left(X-\mu \right)}\right\}$$

Nevertheless, the dynamic and ever-changing nature of the indoor environment poses challenges for assuming a normal distribution of received signal strengths collected from grid points. The study aimed to measure signal strength variations over time for adaptive long-term Wi-Fi indoor localization using RSS fingerprinting. To gain a comprehensive understanding of the datasets and form meaningful hypotheses, an initial data preprocessing step was carried out. This provided valuable insights and a solid foundation for the research. The analysis focused on examining the importance of all signal characteristics gathered throughout the study duration. Three techniques were employed:

(i) Selection of significant signal features based on their mean values.
(ii) Application of FDA and PCA to extract essential features.
(iii) Leveraging positive knowledge transfer into the target domain to enhance indoor location performance.

These methods were used to address the significant concerns related to effectively modeling the problem at hand, given the complex indoor environment. To address the challenges posed by the dynamic indoor environment, the proposed algorithm leverages a new feature space derived from the offline source fingerprints as depicted in Figure 3. This derived fingerprint database was constructed during the training phase following a data preprocessing step to scrutinize the dataset and mitigate the effects of the indoor environment. This involved identifying and addressing outliers and noisy measurements. The derived source domain and the offline fingerprint database were then combined to build a new feature space. This integrated feature space is hypothesized to improve positioning performance in the target domain. Classifiers were then trained using the newly derived feature spaces, and predictions were made based on the newly received fingerprints. By constructing this enhanced feature space, the proposed algorithm aims to overcome the limitations of the original indoor environment and provide more robust and accurate indoor positioning capabilities. The ⊕ symbol used below represents the data integration.

### 3.1. Functional Discrimanat Analysis

The goal of FDA, also called Fisher’s linear discriminant analysis (FLDA), is to discriminate different classes in low dimensional space by retaining the components containing feature values that have the best separation across classes. It is basically to identify the best projection subspace for a specific training sample set such that the projection points of similar samples are clustered in this projection subspace whereas the projection points of various sample types are dispersed. As a result, it may guarantee that the training sample set after projection has the greatest possible interclass distance and the smallest possible intraclass distance in the new subspace. LDA projects features from higher dimension to lower dimension space, and below are the steps for how LDA achieves the goal of discrimination.

1. Computes mean vectors of each class of dependent variable

Recall that we have L different reference points or classes with a total of n signal strength measurements received from the available Wi-Fi access points (APs). Table 1 presents the list of notations and their descriptions used in this study. We define the $n\times p_{s}$ offline source data matrix as follows:

(4) $$R_{im}^{\left(k\right)}={\left[r_{im}^{\left(0\right)},r_{im}^{\left(1\right)},\ldots ,r_{im}^{\left(L-1\right)}\right]}^{T};\;i=1,2,\ldots ,n_{k}\;\&\;m=1,2,\ldots ,p_{s}\;$$

A reference point k has $n_{k}$ RSS measurement samples calculated as follows:

(5) $$r_{im}^{\left(k\right)}{|}_{k=0}^{L-1}\in R^{d};i=1,2,\ldots ,n_{k}\&m=1,2,\ldots ,p\;\mathrm{and}\;n=\sum_{k=0}^{L-1}n_{k}$$

The average RSS measurement values of a reference point k is given as follows:

(6) $${\bar{r}}_{k}=\frac{1}{n_{k}}\sum_{i=0}^{n_{k}}r_{im}^{\left(k\right)}$$

and similarly, the grand mean of all the measurements of the defined region L is given as follows:

(7) $$\bar{R}=\frac{1}{L}\sum_{k=0}^{L-1}{\bar{r}}_{k}$$

2. Computes within-class and between-class scatter matrices.

The scatter matrix between class and within class are given as follows:

(8) $$S_{B}=\sum_{k=0}^{L-1}n_{k}\left({\bar{r}}_{k}-\bar{r}\right){\left({\bar{r}}_{k}-\bar{r}\right)}^{T}$$

(9) $$S_{w}=\sum_{m=1}^{p}\sum_{k=0}^{L-1}\sum_{\;i\;\in \;class\;i}\left(r_{im}^{\left(k\right)}-{\bar{r}}_{k}\right){\left(r_{im}^{\left(k\right)}-{\bar{r}}_{k}\right)}^{T}$$

3. Computes eigen values and eigen vectors for the scatter matrix within class $\left(S_{w}\right)$ and scatter matrix between class $\left(S_{B}\right)$.
4. Sorts the eigen values in descending order and select the top λ.
5. Creates a new matrix containing eigenvectors that map to the λ eigenvalues.
6. Obtains the new features (i.e., linear discriminants) by taking the dot product of the data and the matrix. Below are the details for the above steps mentioned in 4 to 6. Specifically, we define the training instances matrix of a reference point k with a dimension of $n_{k}\times p$ as $r_{im}^{\left(k\right)}{|}_{k=0}^{L-1}={\left[r_{1m}^{\left(k\right)},r_{2m}^{\left(k\right)},\ldots ,r_{n_{k}m}^{\left(k\right)}\right]}^{T};m=1,2,\ldots ,p$. Suppose that we have L reference points or categories instead of just only binary classes or outputs. We are now seeking $\left(L-1\right)$ projection $\left[y_{1},y_{2},\ldots ,y_{L-1}\right]$ by means of $\left(L-1\right)$ projection vectors $w_{i}$. The $w_{i}$ values can be arranged by columns into a projection matrix as $W=\left[w_{1}|w_{2}|\ldots |w_{L-1}\right]$ such that the following is true:

(10) $$y_{i}=w_{i}^{T}x\Rightarrow \;y=W^{T}x$$

where

$$x_{n\times 1}={\left[r_{1},r_{2},\ldots ,r_{n}\right]}^{T},\;y_{L-1\times 1}={\left[y_{1},y_{2},\ldots ,y_{L-1}\right]}^{T}\;\mathrm{and}\;W_{1\times L-1}=\left[w_{1}|w_{2}|\ldots |w_{L-1}\right].$$

Thus, if we have p feature vectors, we can stack them into one matrix as follows:

(11) $$Y=W^{T}X$$

where

$$X_{n\times p}=\left[\begin{array}{c} r_{1}^{1}\\ \vdots \\ r_{n}^{1} \end{array}\begin{array}{c} r_{1}^{2}\\ \vdots \\ r_{n}^{2} \end{array}\begin{array}{c} \cdots \\ \vdots \\ \cdots \end{array}\begin{array}{c} r_{1}^{p}\\ \vdots \\ r_{n}^{p} \end{array}\right],\;Y_{L-1\times p}=\left[\begin{array}{c} y_{1}^{1}\\ \vdots \\ y_{L-1}^{1} \end{array}\begin{array}{c} y_{1}^{2}\\ \vdots \\ y_{L-1}^{2} \end{array}\begin{array}{c} \cdots \\ \vdots \\ \cdots \end{array}\begin{array}{c} y_{1}^{p}\\ \vdots \\ y_{L-1}^{p} \end{array}\right]$$

and $W_{p\times L-1}=\left[w_{1}|w_{2}|\ldots |w_{L-1}\right]$. 

Recalling the two-classes case, the within-class scatter and between scatter were computed as follows:

(12) $$S_{w}=S_{1}+S_{2}$$

(13) $$S_{w}=\sum_{k=0}^{L-1}S_{k}\;\mathrm{where}\;S_{k}=\sum_{k=0,x\in \omega_{i}}^{L-1}\left(x-{\bar{r}}_{k}\right){\left(x-{\bar{r}}_{k}\right)}^{T}\;\mathrm{and}\;{\bar{r}}_{k}=\frac{1}{n_{k}}\sum_{i=0}^{n_{k}}r_{im}^{\left(k\right)}$$

(14) $$S_{B}=\left({\bar{r}}_{1}-{\bar{r}}_{2}\right){\left({\bar{r}}_{1}-{\bar{r}}_{2}\right)}^{T}$$

Similarly, this can be generalized for the C-categories or reference points case as follows. For multiclass classification problem or L reference points case, we will measure the between-class scatter with respect to the mean of all classes as given below:

(15) $$S_{B}=\sum_{k=0}^{L-1}n_{k}\left({\bar{r}}_{k}-\bar{r}\right){\left({\bar{r}}_{k}-\bar{r}\right)}^{T}$$

where $\bar{r}=\frac{1}{n}\sum_{m=1}^{p}\sum_{k=0}^{L-1}\sum_{i=0}^{n}r_{im}^{\left(k\right)};$ ${\bar{r}}_{k}=\frac{1}{n_{k}}\sum_{i=0}^{n_{k}}r_{im}^{\left(k\right)};$ and n and $n_{k}$ represent the number of all data and number of RSS measurements in a reference point of k. Similarly, we can define the mean vectors for the projected measurements y as $\bar{r}=\frac{1}{n}\sum_{m=1}^{p}\sum_{k=0}^{L-1}\sum_{i=0}^{n}y;$ ${\bar{r}}_{k}=\frac{1}{n_{k}}\sum_{i=0}^{n_{k}}y$, where n and $n_{k}$ number of all data and number of data samples in reference point k. Thus, the scatter matrices of both the within class and between classes for the projected samples y are be given as follows:

(16) $${\tilde{S}}_{w}=\sum_{k=0}^{L-1}{\tilde{S}}_{i}=\sum_{k=0}^{L-1}\sum_{y\in \omega_{i}}\left(y-{\bar{r}}_{ki}\right){\left(y-{\bar{r}}_{k}\right)}^{T}$$

(17) $${\tilde{S}}_{B}=\sum_{k=0}^{L-1}n_{k}\left({\bar{r}}_{k}-\bar{r}\right){\left({\bar{r}}_{k}-\bar{r}\right)}^{T}$$

Recall that the binary classification case, we have expressed the scatter matrices of the projected samples in terms of those of the original samples as follows:

(18) $${\tilde{S}}_{W}=W^{T}S_{W}W$$

(19) $${\tilde{S}}_{B}=W^{T}S_{B}W$$

Which still hold for multiple classification problem or L reference points case. Recall that we are looking for a projection that maximizes the ratio of between-class to within-class scatter. Since the projection is no longer a scalar (it has L−1 dimensions), we then use the determinant of the scatter matrices to obtain a scalar objective function as follows:

(20) $$J\left(W\right)=\frac{\left|{\tilde{S}}_{B}\right|}{\left|{\tilde{S}}_{W}\right|}=\frac{W^{T}S_{B}W}{W^{T}S_{W}W}$$

And we will seek the projection $W^{*}$ that maximizes this ratio. To find the maximum of J(W), we differentiate with respect to W and equate to zero. Recall that in the two-classes case, we solved the eigen value problem.

(21) $$S_{W}^{-1}S_{B}w=\lambda w\;\;\mathrm{where}\;\lambda =J\left(w\right)=scalar$$

Similarly, for L reference points, we have L−1 projection vectors, hence the eigen value problem can be generalized to the L-labels as follows:

(22) $$S_{W}^{-1}S_{B}w_{i}=\lambda w_{i}\;\mathrm{where}\;\lambda =J\left(w_{i}\right)=scalar,i=0,1,\ldots ,L-1$$

Thus, it can be shown that the optimal projection matrix $W^{*}$ is the one whose columns are the eigenvectors corresponding to the largest eigen values of the following generalized eigen value problem:

(23) $$S_{W}^{-1}S_{B}W^{*}=\lambda W^{*}\;$$

where $\lambda =J\left(W^{*}\right)=scalar$ and $W^{*}=\left[w_{0}^{*}|w_{1}^{*}|\ldots |w_{L-1}^{*}\right]$.

### 3.2. Principal Component Analysis

In the context of heterogeneous transfer learning for indoor positioning, two main challenges have been identified. Firstly, the assumption of independence among Wi-Fi signals received at a grid point from multiple APs can be problematic, as the RSS values may contain duplicates, leading to interference and the generation of irrelevant features or patterns in the database. Secondly, differences in the dimensions of the source feature spaces and target domains pose challenges when implementing Wi-Fi RSS fingerprint-based techniques, as the dynamic nature of RSS value spread, and the inherent heterogeneity of hardware devices make it difficult to represent signal fluctuations with a single value for a specific position. To enhance indoor positioning performance using RSS fingerprinting, the research focuses on reducing computational complexity and cost through the application of PCA [81,82,83] and FLDA using transfer learning methods [87,90]. The objective is to generate new feature spaces that retain only the most significant predictors, improving model separability and indoor positioning estimates. The algorithm aims to be computationally efficient and cost-effective.

The proposed feature selection-based PCA algorithm (Algorithm 1) utilizes PCA to create a new fingerprint feature space with reduced dimensions [87]. The algorithm employs data preprocessing techniques to decrease the dimensionality of RSS measurements in the offline fingerprint database based on the contribution of features to positioning performance. Features with a higher explainability variance ratio, which are more significant in discriminating the model’s positioning performance, are selected and retained for further analysis. During the testing phase, the learned model is applied after the PCA data preprocessing step to infer the location of the mobile user. The feature selection-based PCA algorithm effectively addresses the challenge of high dimensionality in a predefined radio map by linearly combining features into an uncorrelated space using the training covariance matrix of the radio map. The fingerprint database is then projected into this uncorrelated space, selecting principal components based on their highest explainability variance ratio, which represents their information content. In this paper, the noise created due to the duplicated fingerprints and interdependence of APs is handled by using both the PCA (as in Algorithm 1) and correlation coefficient techniques, which could decrease the dependency of certain Wi-Fi APs or extract the most significant fingerprint features that could be used to build homogeneous feature spaces. The Wi-Fi signals received at a reference point from multiple independent APs can be defined as follows:

(24) $$r_{im}^{\left(k\right)}=\overset{n_{k}}{\underset{i=1}{\cup }}r_{im}^{\left(k\right)};\;k=0,1,\ldots ,L-1,m=0,1,\ldots ,p-1,\;i=1,\ldots ,n$$

(25) $$\mathrm{And}\;P\left(\overset{p-1}{\underset{m=0}{\cap }}r_{im}^{\left(k\right)}\right)=P\left(\overset{n}{\underset{i=1}{\cup }}r_{i0}^{\left(k\right)}\right).P\left(\overset{n}{\underset{i=1}{\cup }}r_{i1}^{\left(k\right)}\right)\ldots P\left(\overset{n}{\underset{i=1}{\cup }}r_{ip-1}^{\left(k\right)}\right)$$

Recall that, the $i^{th}$ Wi-Fi signal strength of fingerprints received at the $k^{th}$ reference point of the $m^{th}$ Wi-Fi AP is a vector denoted as $r_{im}^{\left(k\right)}$ and the fingerprint database collected over the defined region of L reference points can be represented as a multi-dimensional matrix written as follows:

$$R_{im}^{\left(k\right)}={\left[r_{i1}^{\left(k\right)},r_{i2}^{\left(k\right)},\ldots ,r_{ip}^{\left(k\right)}\right]}^{T}={\left[x_{i1}^{\left(k\right)},x_{i2}^{\left(k\right)},\ldots ,x_{ip}^{\left(k\right)}\right]}^{T};\;i=1,2,\ldots ,n_{k}\;\&\;m=1,2,\ldots ,p\;.$$

The correlation coefficient of the RSS measurements of the Wi-Fi access points can be determined as follows:

(26) $$\rho_{r_{1}r_{2}}=\frac{\mathrm{cov}\left(r_{m},r_{m+1}\right)}{\sqrt{\mathrm{var}(r_{m})\mathrm{var}(r_{m+1})}}$$

where $\mathrm{cov}\left(x_{m},x_{m+1}\right)$ denotes the covariance of $x_{m}$ and $x_{m+1}$, which is given as follows:

(27) $$\mathrm{cov}\left(r_{m},r_{m+1}\right)=\frac{\sum \left(r_{m}-{\bar{r}}_{m}\right)\left(r_{m+1}-{\bar{r}}_{m+1}\right)}{n-1}$$

The variances of the measurements $x_{m}$ and $x_{m+1}$ can be computed as follows:

(28) $$\mathrm{var}\left(r_{m}\right)=\frac{\sum_{i=1}^{n_{m}}{\left(r_{im}-{\bar{r}}_{m}\right)}^{2}}{n_{m}-1}\&\;\mathrm{var}\left(r_{m+1}\right)=\frac{\sum_{i=1}^{n_{m+1}}{\left(r_{i\left(m+1\right)}-{\bar{r}}_{m+1}\right)}^{2}}{n_{m+1}-1}$$

where the sample means of the CSI amplitude for the random vectors of Z and Q can be given as follows:

(29) $${\bar{r}}_{m}=\frac{1}{n_{m}}\sum_{i=0}^{n_{m}}r_{im}^{\left(k\right)}\;\mathrm{and}\;{\bar{r}}_{m+1}=\frac{1}{n_{m+1}}\sum_{i=0}^{n_{m+1}}r_{i\left(m+1\right)}^{\left(k\right)}$$

Thus, the PCA algorithm has four major steps:

(1) Standardize each RSS value as the following:

(30) $$z_{im}^{\left(k\right)}=r_{im}^{\left(k\right)}-\frac{1}{n_{m}}\sum_{i=0}^{n_{m}}r_{im}^{\left(k\right)}$$

(2) Calculate covariance matrix of the RSS sample measurements:

(31) $$\sum_{i=1}^{n}\left(r_{im}^{\left(k\right)}-{\bar{r}}_{m}\right){\left(r_{im}^{\left(k\right)}-{\bar{r}}_{m}\right)}^{T}=XX^{T}$$

(3) Eigen value decomposition of covariance matrix;
(4) Obtain projection matrix.

Finally, Algorithm 1 below is a pseudocode to construct the refined source domain based on mean signal values and multi-criteria feature extractions (LDA-CA-PCA). On the other hand, the proposed algorithm comprises two primary phases. Firstly, in the training phase, RSS fingerprints are gathered from the available Wi-Fi APs at defined RPs. This entails sampling the signals from the APs at the RPs and storing the RSS measurements. Secondly, in the testing phase, the algorithm utilizes the collected fingerprints to estimate the location of a mobile device. It accomplishes this by comparing the received RSS measurements from the mobile device with the stored fingerprints in the database. Through techniques like pattern matching or machine learning algorithms, the algorithm determines the most probable location of the mobile device based on the similarity of the RSS measurements to the stored fingerprints.

**Algorithm 1.** Construction of refined source domain based on simulated signal parameters and multi-criteria feature extractions (LDA-CA-PCA)**Input:** (1) Offline database $R_{tr}$; (2) Testing domain $R_{ts}$; (3) Simulation signal parameters; (4) # of testing instances $n_{t}$ **Output:** Refined source domain $R_{S}$ 1. $R_{S}=\left[\;\right]$ 2. **for** k=0,1,…,L−1 **do** 3.   **for**
m=1,2,…,p **do** 4**.**    **for**
$i=1,2,\ldots ,n_{tr}$ **do** 5.     Apply data processing (24–31): 6.    —Check outliers and normality of the measurements using histograms and boxplot 7.    —Test heteroscedatiy of the measurements using histograms 8.    —Establish linearity of the parameteres 9.    **end for** 10.    Generate feature spaces based on mean signal values as in Equations (29) and (30) 11.    Build refined source domain based on multi-criteria feature extractions 12.    Apply LDA to extract features with higher class separabiliy as in Equations (4)–(22) 13.    Apply PCA and CA to extract features with sigifiacnt predictord as in Equations (24)–(31) 14.   **end for** 15. **end for** 16. **for**
$i=1,2,\ldots ,n_{S}$ **do** 17.  $x_{of}^{p}\to x_{S}^{p_{s}},y_{of}^{p}\to y_{S}^{p_{s}}$ 18.  $R_{S}=\left\{\left(x_{S}^{p_{s}},y_{S}^{p_{s}}\right)\right\}$ 19. **end for** 20. return $R_{S}$

To tackle the challenges that have been identified in the context of heterogeneous indoor positioning, a data integration approach has been proposed to leverage the transfer of heterogeneous knowledge from the related source domain to the target domain. The objective of this approach is to improve indoor positioning performance by combining information from diverse domains. The pseudocode for the proposed knowledge transfer based on mean signal parameters and hybrid feature extractions (LDA-CA-PCA) for indoor positioning is provided in Algorithm 2.

**Algorithm 2.** Proposed knowledge transfer based on mean signal values and hybrid feature extractions (LDA-CA-PCA) for indoor positioning**Input:** (1). Refined Training data $R_{S}$; (2) Testing data $R_{ts}$; (3) # of instances $n_{s}$, $n_{t}$; **Output:** 1. Domain mapping of $R_{S}$, $R_{T}$; 2. Projection matrix $W^{*}$; 3. Target labels $y_{T}$; 1. **for** k=0,1,…,L−1 **do** 2**.**    **for** m=1,2,…,p **do** 3.      **for**
$i=1,2,\ldots ,n_{tr}$ **do** 4.          Reuse steps from 5–8 of Algorithm 1 5.          Apply LDA techniques as detailed in (4–22) 6.       **end for** 7.       Make selection on optimal refined features using hybrid matrices 8.    **end for** 9. **end for** 10. **for** k=0,1,…,L−1 **do** 11.    **for**
m=1,2,…,p **do** 12**.**       **for**
$i=1,2,\ldots ,n_{ts}$ **do** 13.       Compute optimal projection matrix $W^{*}$ applying Equations from (16)–(23) 14.       **end for** 15.    **end for** 16. **end for**
17. Train a classifier on $R_{S}$ and $y_{S}$ and optimize projection matrix of source samples $W^{*}$ 18. Estimate $y_{T}$ on $R_{T}$ by applying the trained classifier $f\left(\left(R_{S},y_{S}\right),W^{*}\right)$ 19. **return**
$R_{S}$,$y_{S}$,$R_{T}$,$y_{T}$,$W^{*}$

### 3.3. Evaluation Metrics for Indoor Positioning Performance

The proposed algorithm for adaptive long-term Wi-Fi indoor localization was evaluated against various baseline machine learning algorithms and other identified states-of-the-arts using extensive real-world datasets. The effectiveness of the localization system was measured using mean absolute error (MAE), which quantifies the average deviation between estimated positions and actual locations, with lower MAE values indicating higher accuracy. The indoor localization problem is often approached as a multiclassification task where different locations are regarded as labels to be classified [89]. It is defined as follows:

(32) $$MAE=\frac{1}{n}\sum_{m=1}^{n}\left[\left|\left({\hat{x}}_{m}-x\right)|+|\left({\hat{y}}_{m}-y\right)\right|\right]$$

where ${\left[{\hat{x}}_{m},{\hat{y}}_{m}\right]}^{T}$ and ${\left[x,y\right]}^{T}$ are the predicted location estimate and the true location of a client of the 2-dimensional coordinates of the $m^{th}$ positioning sample, respectively. And n is the total number of samples to be located in the target domain.

## 4. Experimental Results and Discussion

### 4.1. Experiment Setup

**Figure 4:**
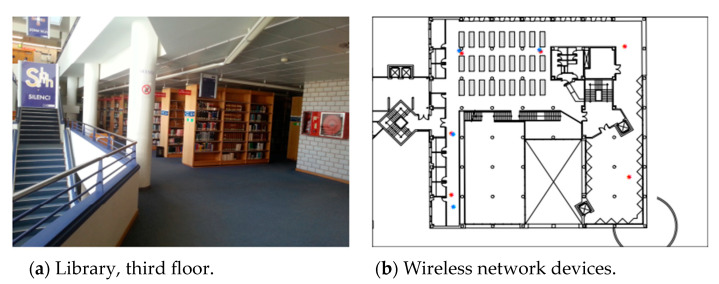
Library environment: (**a**) a picture of the third-floor collection area that shows the bookshelves and the stairs that connect the two floors; and (**b**) the network devices close to the collection area. The red asterisks represent the third floor’s devices, and blue asterisks represent fifth floor’s devices [90].

The study was conducted at the Universität Jaume I library in Spain, specifically on the 3rd and 5th floors, as shown in Figure 4a, covering an area of approximately 308.4 m^2^. Over a period of 25 months, a total of 620 Wi-Fi APs were deployed, as shown in Figure 4b. 

The measurements were collected using a Samsung Galaxy S3 smartphone smartphone (https://zenodo.org/records/1066041 accessed on 15 July 2024) by a single individual. In the first month, fifteen offline databases and five online databases were collected, and in the subsequent months, one offline database and five online databases were collected using the same smartphone. The 15 offline databases from the first month were chosen as the offline database, while the online databases from each subsequent month were used as testing samples. All of these databases can be accessed at https://zenodo.org/record/1066041#.W0wHdfknYYJ (accessed on 21 January 2024). The collection process involved predefined positions and the collection of six fingerprints at each position. The collected measurements were uploaded to a server to create labeled training and test datasets. The collection period was divided into 25 collection months, with varying numbers of training and test datasets. In our experimental setup, we designated the four offline databases from the first month as our reference database, and we used all the online databases from each subsequent month as the testing samples to investigate fluctuations in signal features or measurements during sampling. Furthermore, we systematically selected a sole training dataset from each month for the purpose of scrutinizing the fluctuations in sample selection spanning multiple months (specifically, four months) in order to assess or estimate long-term indoor localization. Subsequently, we subjected this dataset to evaluation against five distinct testing samples to gauge the variability in signal characteristics within the samples gathered over randomly chosen months. For more detailed information, refer to reference [90]. The long-term Wi-Fi fingerprinting dataset and supporting material are available on Zenodo. In Table 2, there is a detailed description of the dataset that was utilized for adaptive long-term Wi-Fi indoor localization. This dataset specifically focuses on using received signal strength as a signal feature and includes 620 features. It was collected over a period of 25 months and consists of a total of 106 grid points.

### 4.2. Exploring Wi-Fi RSS Distribution Characteristics

**Figure 5:**
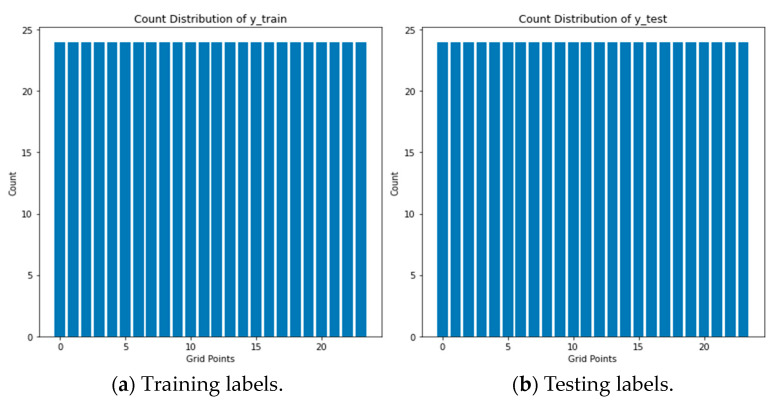
Comprehensive examination of target variable distribution of measurements in both datasets.

In this section, the main focus is on the preprocessing of Wi-Fi RSS measurement data in order to understand various characteristics of the dataset. These characteristics include the presence of outliers, the central values of the distribution, the spread of the data, the skewness of the distribution, and the normality of the data. These factors can potentially present significant challenges for the performance of algorithms used in indoor positioning systems. The ensuing bar plots of Figure 5 provided a comprehensive examination of the target variable distribution across diverse grid points. These visualizations serve to elucidate the frequency and prevalence of each grid point within both the training and testing datasets. An additional noteworthy observation arises from scrutinizing the distribution of signal measurements per reference points (RPs) for both training and testing labels. The discernible equality in the number of measurements taken from each grid point contributes to the establishment of balanced target labels. This, in turn, signifies a deliberate mitigation of potential adverse effects on indoor location predictions stemming from issues associated with data imbalance—a phenomenon that, fortuitously, is absent in this particular dataset.

**Figure 6:**
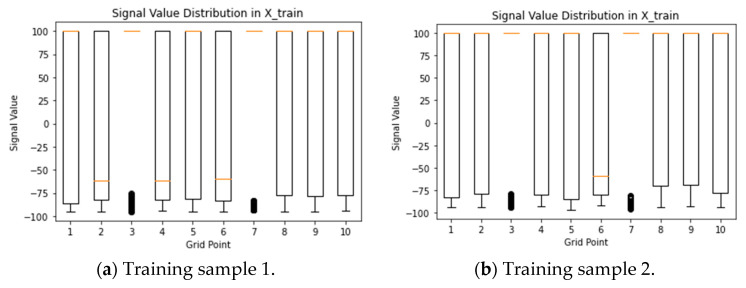

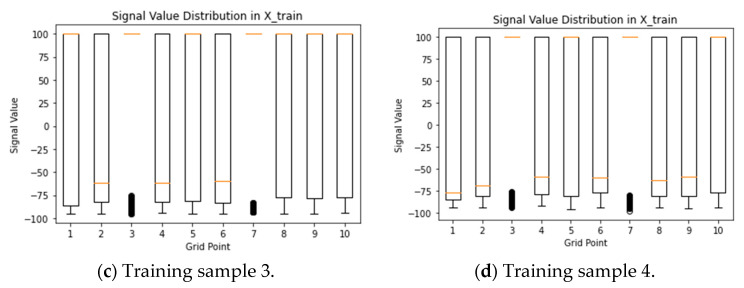
Box-plot distribution of signals for selected GPs of training samples from month 1.

**Figure 7:**
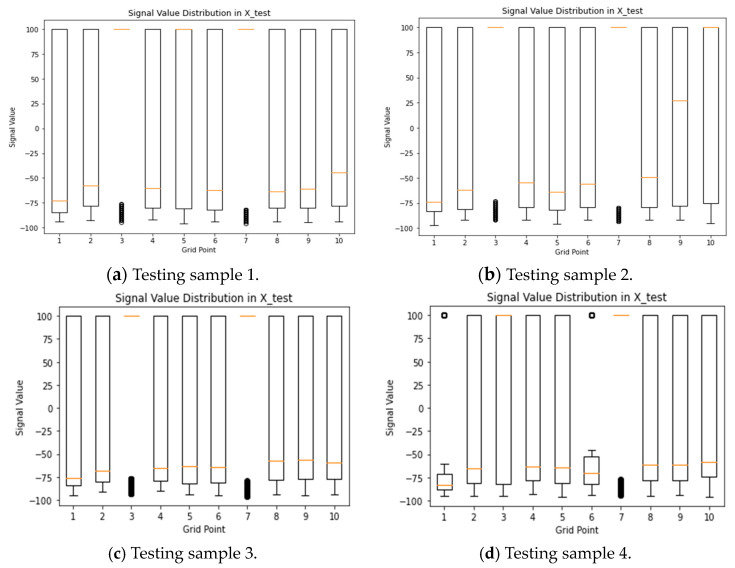
Box-plot distribution of signals for selected GPs of testing samples from month 1.

The box plots of Figure 6 and Figure 7 below provide a visual representation of the distribution of the signal values for the selected grid points in both the training (X_train) and testing (X_test) datasets. The box plots depict signal values across grid points, with the x-axis representing grid points and the y-axis indicating signal values’ range and spread. Position on the y-axis reflects signal values’ magnitude. Box plots aid in outlier identification, data spread assessment, and signal value distribution comparison. Examining these plots provides insights into signal characteristics and variations within the selected grid points in training and testing datasets. Each box plot represents one grid point, and the box itself represents the interquartile range (IQR) of the data, which encompasses the middle 50% of the values. The line inside the box represents the median value, and the lines extending from the box (whiskers) represent the range of the data within a certain threshold. Any data points outside this threshold are considered outliers and are plotted individually as points. By examining the box plots, you can compare the distribution of signal values between the training and testing datasets. Since the box plots for the same grid point of 2, 4, 5, and 6 in both datasets of (month 1 and testing sample 1) have similar shapes and positions, it suggests that the distribution of signal values for this grid point is consistent across the datasets. On the other hand, the box plots differ significantly for 1, 8, 9, and 10, which indicates differences in the signal value distribution between the datasets. 

One can observe a cluster of dots instead of a visible box plot for grid points of three and seven; it typically indicates that there may be outliers in the data for those specific grid points. The box plots below are used to visually represent the distribution of data, and they include components such as the median, quartiles, and potential outliers. When you see a cluster of dots without a visible box, it suggests that there are many outliers in the data for that specific grid point. Outliers can significantly affect the appearance of the box plot, and in extreme cases, the whiskers may be too short to show the spread of the majority of the data. We suggest conducting a detailed examination of the actual values corresponding to grid points three and seven. We assess the validity of these values and investigate for potential issues in the data, such as errors or anomalies. Furthermore, we considered adjusting the plotting parameters or exploring alternative visualization techniques to gain a clearer understanding of the data distribution for these specific grid points. However, most recorded values on these grid points are consistently set to 100, indicating no signal measurement was detected at those specific grid points. 

**Figure 8:**
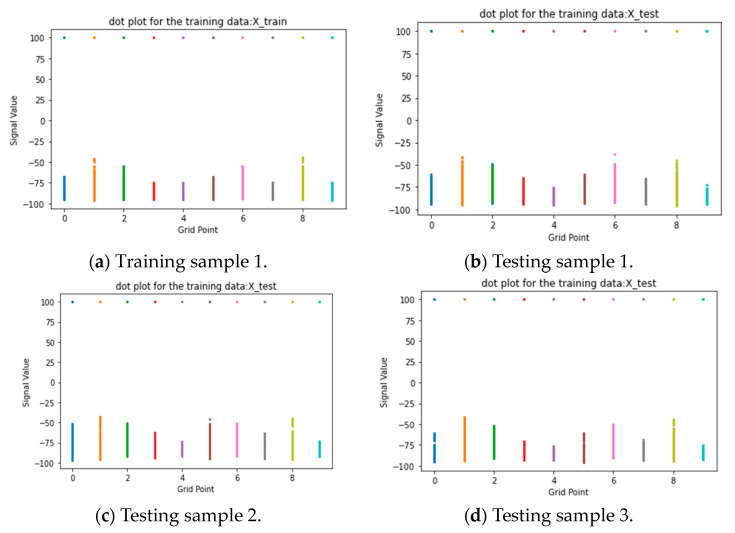
Signal value distribution of selected grid points of both samples from month 1.

Figure 8 explores alternative visualization techniques to better understand the data distribution for specific grid points in both the training and testing samples of one collected in month 1. The figure includes a boxplot and a dot plot representing the signal value distribution of the selected grid points, which align with each other. Additionally, the majority of recorded values at these grid points consistently show a value of 100, signifying the absence of signal measurements at those specific locations.

**Figure 9:**
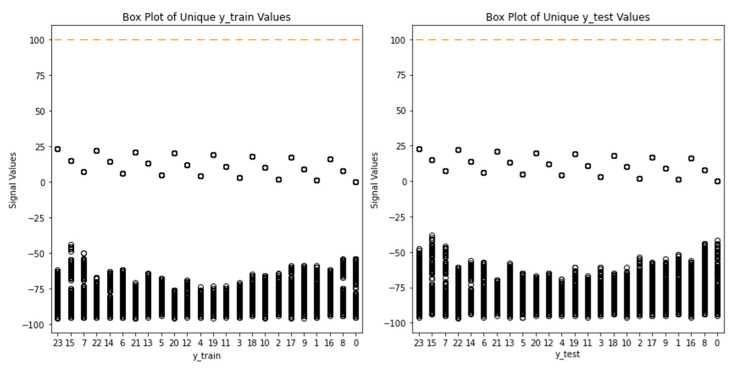
Distribution of received signal values by unique GPs: training vs. testing data.

Figure 9 illustrates the distribution of measurements for the unique values of ‘y_train’ and ‘y_test’ in the training and testing samples of month 1. By comparing the box plots of ‘y_train’ and ‘y_test’, we can visually analyze and compare how the signal values are distributed for each category in the training and testing datasets. The box plots show that the signal measurements collected at a specific grid point are diverse and cover a wide range of received signal strength. Additionally, the box plots indicate the presence of outlier values for almost all target labels.

**Figure 10:**
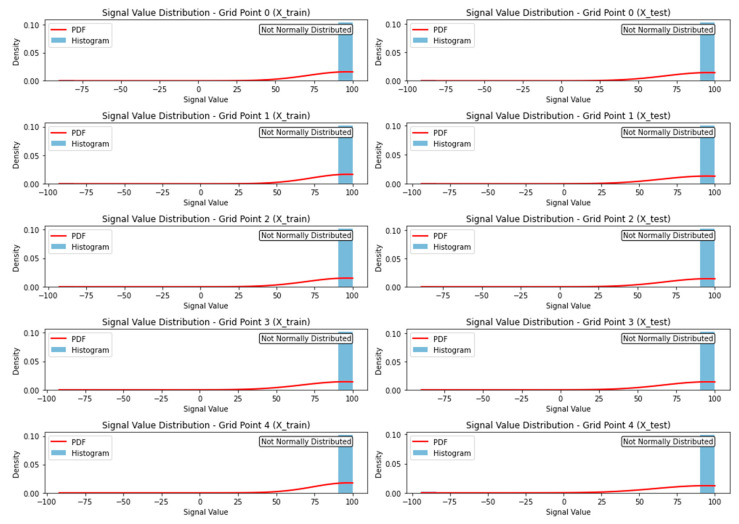
Signal value distributions and normality test results for GPs in X_train and X_test.

Figure 10 analyzes and visualizes the distribution of signal values for different grid points in the X_train and X_test datasets. It employs the Shapiro-Wilk test to assess whether the signal values follow a normal distribution. The distribution is represented through histograms, while the PDFs fit normal distribution curves to the data. The Shapiro-Wilk test is conducted on the signal values of each grid point in both datasets, comparing the resulting p-values to a significance level of 0.05. The normality status (“Normally Distributed” or “Not Normally Distributed”) is indicated alongside each plot. The interpretation involves examining the histograms, PDFs, and normality test results to gain insights into the shape and characteristics of the signal value distributions. The findings suggest that the specified grid points in the training and testing datasets do not exhibit a normal distribution. This has implications for adaptive long-term indoor localization, as the diverse distribution of signal values poses a challenge for establishing a universal model. Traditional localization algorithms assuming normality may not be effective in this context. Therefore, robust and adaptive techniques, such as machine learning algorithms, are needed to handle the diverse signal value distribution and improve the accuracy of indoor localization.

**Figure 11:**
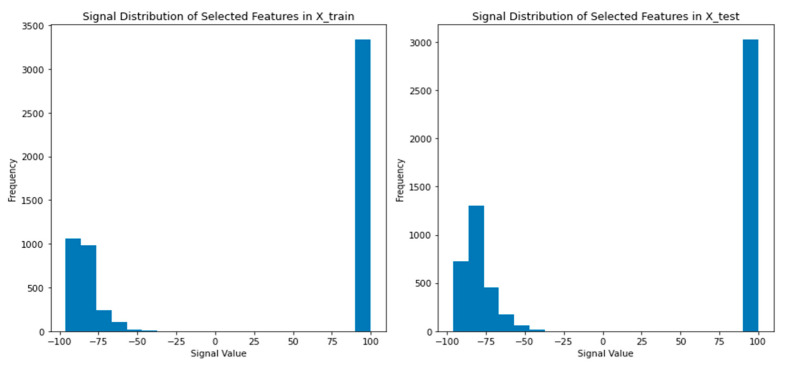
Signal Distribution Comparison between training and testing sample 3, Month 1.

Figure 11 illustrates the signal distribution comparison between training and testing sample 3 from month 1. It compares the signal distribution of selected features in the training and testing datasets. The Mann–Whitney U test confirms a significant difference between the distributions, indicating the dynamic nature of the indoor environment. This finding suggests that models trained on the training dataset may face challenges when applied to the test dataset. To improve performance, it is important to develop adaptable models that can account for variations in the indoor environment.

**Figure 12:**
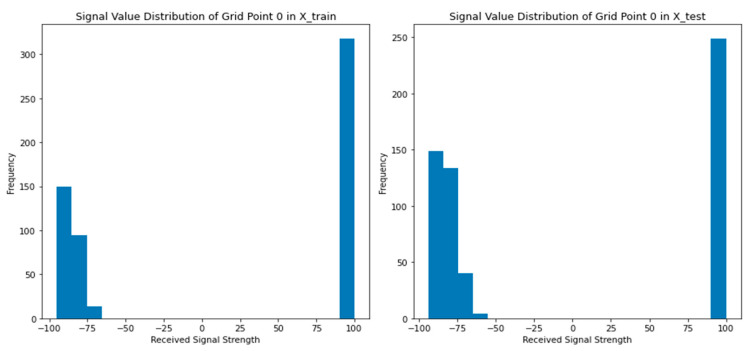
Signal distribution comparison of Grid point 0 for both training and testing samples.

Figure 12 illustrates the distribution of received signal strength for a specific grid point in both the training set (‘X_train’) and the test set (‘X_test’). The histograms provide a visual representation of the frequency or count of different received signal strength values within specified bins. By comparing the histograms, we can observe the shape, concentration, and position of the bars, which correspond to the frequency of received signal strength values falling within each bin. Additionally, a statistical test (the two-sample Kolmogorov-Smirnov test) was performed to determine whether the distributions in the training and test sets are likely the same or different. Accordingly, the distributions of the received signal strength in training and testing datasets are likely different as the p-value obtained from the test is smaller than 0.05. This result suggests that the received signal strength distribution may have changed or varied between the training and test sets. Thus, the figure and the accompanying statistical test provided insights into the diversity of the received signal strength distribution for the selected grid point between the training and test sets.

### 4.3. Comparative Analysis of Methods

In this section, we conducted a comprehensive set of real-life experiments spanning a period of 25 months. The experiments aimed to measure the temporal fluctuations in signal strength for adaptive long-term Wi-Fi indoor localization, utilizing received signal strength fingerprint measurements. To gain a deep understanding of the datasets and generate meaningful hypotheses regarding adaptive Wi-Fi indoor localization, we performed initial data preprocessing. This preprocessing step allowed us to extract valuable insights and develop a solid foundation for our research. Thus, our analysis focused on four main issues: 

1. Exploring the significance of all signal features collected over the time period of study: We examined whether the signal features played a crucial role in modeling the problem. To address this issue, we employed three different techniques as described in Section 3.

2. Investigating the effects of sampling signal fluctuations over time:

- We examined the impact of sampling signal fluctuations on different algorithms in indoor localization scenarios. Multiple training samples were used to assess the influence of sampling fluctuations, while all collected testing samples for each month were used to evaluate algorithm robustness.
- The research question regarding the vulnerability of algorithms to sampling fluctuations has been explored and investigated, as it is an intriguing area of research.
- We demonstrated the challenges posed by the indoor environment through an analysis of multiple training samples. Additionally, we verified the dynamic nature of indoor positioning by conducting multiple sample testing.
- By employing multiple training and testing samples, we can validate the challenges faced in indoor localization scenarios.

3. Finally, we performed a comparative analysis of the proposed algorithm for adaptive long-term Wi-Fi RSS-based indoor localization. In this study, we conducted a comprehensive comparison of our proposed algorithm, Ada-LT IP, against various machine learning algorithms commonly employed in prediction tasks. Additionally, we performed a comparative analysis with several recent robust algorithms to assess the reliability and robustness of Ada-LT IP in dynamic indoor environments. Specifically, we compared our algorithm to robust indoor positioning [90], LSTP [91], and TransLoc [92].

#### 4.3.1. Exploring the Significance of Signal Features Collected over the Time Period of Study

**Figure 13:**
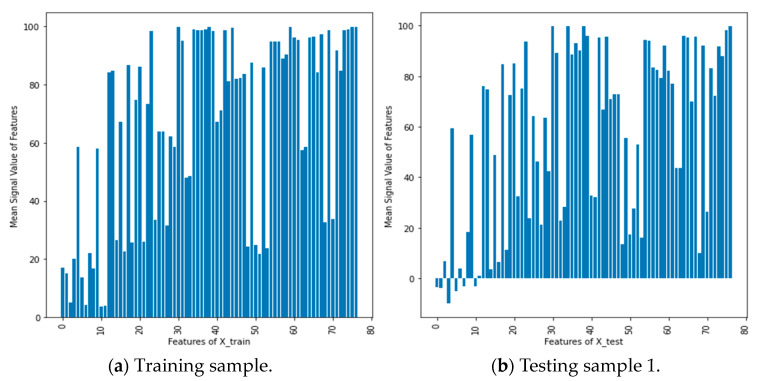
Mean received signal values of Wi-Fi feature spaces for both samples from month 1.

We scrutinized the importance of Wi-Fi feature spaces implemented in the experimental area, where localization can be accomplished by studying the fingerprints containing recorded measurements at predefined positions. During a span of 25 months, a total of 620 Wi-Fi APs was deployed, as illustrated in Figure 4b. However, not all measurements collected from these Wi-Fi APs hold equal significance, and some may even be irrelevant for modeling the indoor localization problem. In light of this, we investigated the mean signal values derived from the received signal measurements obtained from each deployed feature space in the experimental area. Figure 13a,b below showcase the mean received signal values of Wi-Fi feature spaces for training and testing samples of Month_1. The analysis of these mean signal values revealed that only 77 Wi-Fi feature spaces contained actual signal measurements, while the remaining Wi-Fi APs were assigned a global constant value of 100. This constant value denotes the absence of received signal measurements, rendering those Wi-Fi APs unsuitable for device localization purposes. The height of each bar corresponds to the mean signal value of a specific feature, and the x-axis represents the feature names. By comparing the bar heights between the two graphs, differences in mean signal values can be observed. These graphs provide a visual representation of the relative magnitudes and potential variations in mean signal values among features in the training and testing datasets, aiding in the analysis and comparison of feature characteristics in the two datasets. Accordingly, 77 significant signal features have been selected for modeling adaptive long-term indoor localization based on their mean values, considering the signal values recorded in each feature.

(a) Wi-Fi Fingerprint-Based Indoor Location Estimation of Targets Utilizing Original Feature Spaces

The impact of Wi-Fi received signal strength fluctuations on the adaptability of indoor localization was evaluated by analyzing multiple training samples collected over a four-month period. Four training samples were randomly selected, and the performance of various classifiers was assessed using five different testing samples from each month. The objective was to assess the robustness of the proposed algorithm in the presence of dynamic signal variations. Table 3 and Table 4 present the performance of the algorithms across different training and testing samples, providing insights into the effects of signal strength fluctuations and the adaptability of the proposed algorithm for long-term Wi-Fi fingerprint-based indoor localization. The results indicate significant variations in localization performance due to the dynamic nature of both training and testing datasets.

(b) Wi-Fi Fingerprint-Based Indoor Location Estimation of Targets Utilizing Derived Feature Spaces based on mean signal strength received values

Table 5 and Table 6 present the implementation of Wi-Fi fingerprint-based indoor location estimation techniques utilizing derived feature spaces, specifically focusing on mean signal strength values from Wi-Fi access points. Our analysis indicates that among the total Wi-Fi feature spaces, only 77 are based on actual signal measurements; the remaining access points are assigned a constant value of 100 (see Figure 13). We also conducted a comprehensive analysis of the algorithm’s performance concerning localization accuracy, the impact of signal strength fluctuations, and adaptability in dynamic indoor environments. These tables provide critical insights into how signal strength variations affect the proposed algorithm’s performance over time. To evaluate the algorithm’s adaptability to signal strength fluctuations, we collected multiple training samples over a 25-month period. Four training samples were randomly selected, and the performance of various algorithms was assessed using five distinct testing samples from each month. This methodology enabled a robust evaluation of the proposed algorithm’s capability to respond to dynamic signal variations. The results demonstrated noticeable variations in localization performance across both training and testing datasets, underscoring the dynamic nature of indoor environments.

#### 4.3.2. Feature Extraction Using Data Reduction Techniques

(a) PCA

In this section, we investigated the impact of feature dimensions on the variance explainability ratio (VER) for indoor location prediction. We conducted the analysis using multiple training samples from the initial month and randomly selected five testing samples to examine the contribution of significant features. Specifically, we examined principal components at various levels or percentages, including PC-80, PC-85, PC-90, PC-95, P-99, and Ref. (which serves as a baseline representing 50% variation in the model, denoted as P-50). Table 7 provides a detailed overview of how feature spaces affect the variance accounted for in Wi-Fi fingerprint-based indoor location estimation for both training and testing sample datasets. We observed that the contributions of feature spaces varied across different testing samples from various months. For example, in the first month’s training sample (#1), P-50 indicated that eight principal components were sufficient for location estimation. However, the corresponding testing sample #1 required 19 principal components for accurate indoor localization. Similar comparisons were made for each proportion of contribution in modeling the localization problem. For instance, P-95 required 50 principal components for the training sample, while the testing samples required 72, 67, 61, 65, and 99 principal components, respectively. These discrepancies in the number of principal components can be attributed to signal fluctuations arising from the indoor environment and the sampling process.

Table 8 provides a comprehensive analysis of the impact of feature spaces on the variance account in Wi-Fi fingerprint-based indoor location estimation. To evaluate this, we utilized several training samples collected over four consecutive months, along with corresponding testing samples from each month. The aim was to assess the contribution of significant features with higher variance explainability ratios by examining principal components (PCs) at various levels or percentages, including PC-80, PC-85, PC-90, PC-95, P-99, and Ref. The detailed results presented in Table 8 outline the effect of feature spaces on the variance account for both training and testing sample datasets in Wi-Fi fingerprint-based indoor location estimation. For instance, P-99 required 67 principal components for the training sample of month #1 (M-1), while the testing samples required 110, 100, 113, and 97 principal components for each month considered (M-1, M-2, M-3, and M-4). The variations in the required number of principal components therefore can be attributed to the influence of signal fluctuations caused by the dynamic nature of the indoor environment and the inherent variability in the sampling process.

Table 9 and Table 10 display the results of the Wi-Fi fingerprint-based indoor location estimation of targets using extracted feature spaces based on principal components. In our study, we examined the impact of feature dimensions on the VER for predicting indoor locations. To conduct this analysis, we utilized multiple training samples from different months and randomly selected five testing samples from each month to assess the contribution of significant features. Specifically, we investigated principal components at different levels or percentages, including PC-80, PC-85, PC-90, PC-95, P-99, and Ref. We evaluated the performance of the proposed algorithm in each case, considering the number of principal components used. Remarkably, our findings consistently indicated that the proposed algorithm outperformed other algorithms across all tested scenarios. It exhibited the best fit in terms of accurately estimating indoor locations, regardless of the specific number of principal components considered. The observed variations in the required number of principal components can be attributed to signal fluctuations originating from the dynamic indoor environment and the inherent variability in the sampling process. These factors influence the optimal selection of principal components needed for effective indoor location estimation.

**Figure 14:**
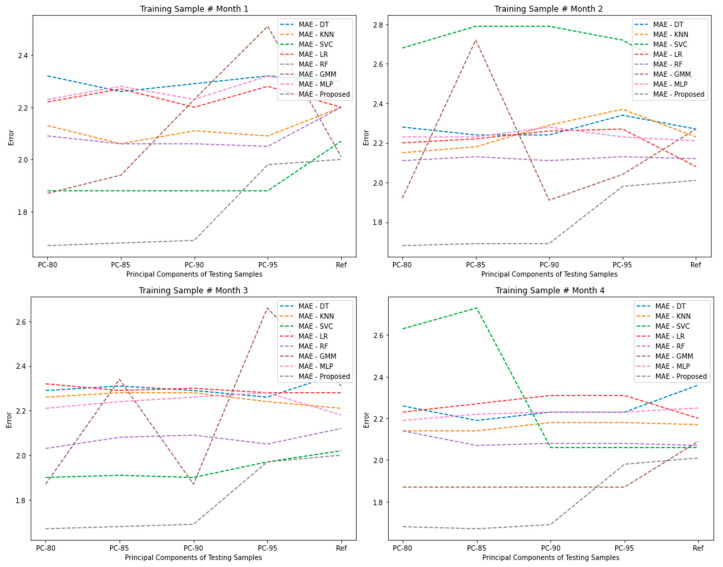
Comparison of localization performance and variance-explained ratio of PCs for different algorithms on testing samples.

Figure 14 illustrates the comparison of MAE and VER of principal components for different algorithms on testing samples. The figure provides a visual representation of the performance of various algorithms in terms of both MAE and the variance explained by the principal components. The x-axis represents the different algorithms being compared, while the y-axis represents the values of MAE and the variance-explained ratio. The figure consists of two sets of bars or lines, one for each metric, corresponding to the different algorithms. The bars or lines for MAE provide a comparison of the absolute error between the predicted values and the actual values for each algorithm on the testing samples. Lower values indicate better performance, as they reflect a smaller discrepancy between the predicted and actual values. The lines or bars representing the variance-explained ratio of principal components illustrate the proportion of variance in the data explained by the principal components. Higher values indicate a greater ability of the principal components to capture the underlying patterns and variability in the data. By visually comparing the bars or lines for each algorithm, one can assess the relative performance in terms of MAE and the ability of the principal components to explain the variance. This comparison helps in understanding the trade-offs between prediction accuracy and the contribution of principal components in capturing the data’s variability for each algorithm on the testing samples. It appears that the proposed algorithm actually has the smallest MAE in both Training Sample # Month 1 and Training Sample # Month 2. In Training Sample # Month 1, the proposed algorithm demonstrates the lowest MAE values across all principal components compared to the other algorithms, including DT, KNN, SVC, LR, RF, GMM, and MLP. Similarly, in Training Sample # Month 2, the proposed algorithm exhibits the lowest MAE values among the compared algorithms. Therefore, results revealed that the proposed algorithm performs the best among the algorithms considered, as it achieves the smallest MAE values for all training sample months.

(b) Functional Discriminant Analysis

In this section, we investigated the impact of feature spaces on the adaptability of Wi-Fi fingerprint-based indoor location estimation. Our aim was to assess the robustness of the proposed algorithm by utilizing various training and testing sample datasets. Table 11 and Table 12 present the results of applying FDA to the estimation of indoor locations based on Wi-Fi fingerprints. Four different training samples were used, each collected over a span of four months (#1, #2, #3, and #4), and their robustness was evaluated against five testing samples collected from each respective month. The results indicate that all algorithms exhibited higher localization errors or poorer localization performance when tested on samples #3 and #4, compared to the remaining testing samples collected across various months. These different samples were specifically chosen to test the adaptability of the algorithms to long-term positioning effects. However, in contrast to this trend, the proposed algorithm consistently demonstrated the lowest MAE, suggesting that it is the most suitable algorithm for indoor localization. Furthermore, the proposed algorithm’s localization performance was evaluated across various training samples and tested against different testing samples. It exhibited the ability to adapt to the evolving indoor environment, as its performance remained superior and yielded the lowest error scores. Therefore, it can be concluded that the FDA extraction feature is effective across different testing samples, despite minor performance variations due to fluctuations in the received signal strength caused by sampling. Hence, it can be inferred that the FDA extraction feature demonstrates efficiency across diverse testing samples, although minor performance variations may arise due to sampling fluctuations in the received signal strength.

#### 4.3.3. Comparison of Localization Performance

This paper presents a comparative analysis of two distinct approaches to Wi-Fi fingerprint-based indoor location estimation: original feature spaces and derived feature spaces utilizing various data reduction techniques: mean signal strength values, PCA, and FDA. 

1. Original Feature Spaces: The evaluation of localization performance across multiple training samples collected over various months revealed significant variations due to temporal signal strength fluctuations. The proposed algorithm, Ada-LT IP, demonstrated robustness in adapting to these dynamic variations, as shown in Table 3 and Table 4.

2. Derived Feature Spaces: Utilizing mean signal strength values (as indicated in Table 5, Table 6, Table 7 and Table 8) and data reduction techniques such as PCA (as indicated in Table 11 and Table 12) and FDA (13 and 14). The analysis identified that only 77 of the Wi-Fi feature spaces are significant with actual signal measurements. The proposed algorithm, Ada-LT IP, demonstrated superior localization accuracy compared to the other algorithms evaluated in the study. Additionally, it proved to be robust in dynamic indoor environments. The study also highlighted discrepancies in the number of principal components required for training versus testing samples, reflecting the influence of signal fluctuations on model performance. Moreover, we performed a comparative analysis with several recent robust algorithms to assess the reliability and robustness of Ada-LT IP in dynamic indoor environments. Despite the inflated error of localization observed in testing and training samples for 3 and 4 due to the inherent significant signal sampling fluctuations, the proposed algorithm outperforms the state-of-the-art performance achieved in robust indoor positioning [90] (all the proposed methods reported their accuracy to be in the range of 2–5 m, LSTP [91] (reported MLE = 2.18 m), and TransLoc [92] (MLE = 2.23 m). The average mean absolute errors of the proposed Ada-LT IP algorithm are significantly lower compared to the state-of-the-art described in the study (as seen in Table 11 and Table 12). Along with this, previous authors have reported that their algorithm (LSTP) outperforms several other algorithms, such as ISMA [93], UFL-ECLS [94], KNN, TCA [95], JGSA [96], CDLS [97], and LPJT [98]. This validation underscores the potential of Ada-LT IP in enhancing indoor positioning accuracy.

3. Adaptability and Feature Extraction: The proposed framework emphasized the importance of feature extraction and adaptability. The use of data reduction techniques illustrated how different feature dimensions affected variance explainability ratios. Intensive experimental results demonstrate that the proposed algorithm maintained superior performance across various training and testing conditions, effectively addressing the challenges posed by a dynamic indoor environment. Thus, the Ada-LT IP algorithm consistently exhibits better adaptability and accuracy in response to signal variability, underscoring the significance of both feature selection and environmental dynamics in effective indoor positioning.

#### 4.3.4. Comparative Analysis of Computational Complexity in Algorithmic Performance

**Figure 15:**
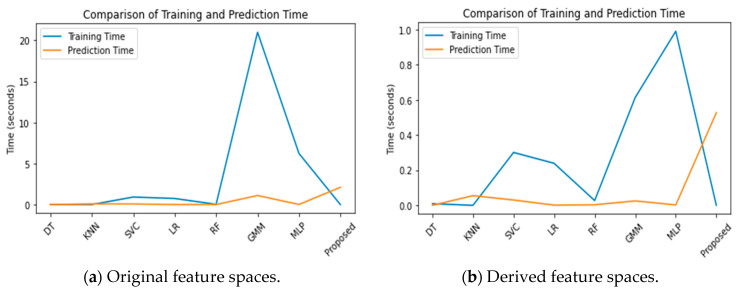
Comparative analysis of algorithm performance based on computational time.

In this study, we investigate the computational complexity of a novel algorithm designed for adaptive long-term Wi-Fi fingerprint-based indoor location estimation. Our analysis emphasizes the effectiveness of various data reduction techniques, including mean signal values, functional discriminant analysis, correlation analysis, and principal component analysis, in extracting feature spaces. The algorithms were executed on a laptop equipped with an AMD Ryzen 3 3200U CPU and 16 GB of RAM. The complexity assessment focuses on both time and space dimensions. Performance comparisons of different algorithms, as shown in Figure 15a, reveal that training times are consistently longer than prediction times across all algorithms, indicating that training requires more computational resources. Notably, despite the higher time complexity, our proposed algorithm achieves superior accuracy in indoor localization compared to others, including GMM and MLP, which exhibit even greater computational demands. Further analysis in Figure 15b contrasts the performance of algorithms using derived feature spaces, which were reduced to 77 dimensions based on mean signal values. This reduction significantly lessens time complexity for all algorithms, enhancing efficiency. Although the proposed algorithm has a slightly longer prediction time than some alternatives, its accuracy justifies this trade-off, establishing it as the optimal choice for indoor localization tasks. Overall, the findings underscore the potential of utilizing reduced-dimensional feature spaces to improve both computational efficiency and accuracy in indoor localization systems.

## 5. Conclusions

This research paper analyzes a comprehensive set of real-life experiments conducted over 25 months to investigate temporal fluctuations in signal strength for adaptive long-term Wi-Fi indoor positioning. The study focuses on the significance of signal features, the effects of sampling fluctuations, and the assessment of dynamic indoor localization using mean absolute error metrics. Techniques such as feature selection based on mean signal values and data reduction methods like PCA and FDA were employed to identify essential features with higher variance explainability ratios at various thresholds (PC-80, PC-85, PC-90, PC-95, P-99, and Ref). Additionally, the research leverages positive knowledge transfer to enhance indoor location performance and evaluates the impact of sampling signal fluctuations on different localization algorithms. Multiple training samples were used to assess these fluctuations, while monthly testing samples evaluated algorithm robustness. The challenges posed by the indoor environment were demonstrated through extensive testing, confirming the dynamic nature of indoor positioning. The proposed Ada-LT IP algorithm integrates data reduction techniques and transfer learning to address offline fingerprint calibration challenges, showing superior accuracy compared to state-of-the-art fingerprint-based positioning methods. The study also addresses multicollinearity through covariance analysis and compares various feature extraction methods. Furthermore, the analysis of computational complexity reveals that utilizing derived feature spaces significantly reduces time complexity across algorithms, enhancing overall efficiency. The findings highlight the potential of Ada-LT IP in improving indoor positioning accuracy and efficiency while tackling challenges related to offline fingerprint calibration and computational complexity.

## Figures and Tables

**Table 1:** List of notations.

Notation	Description
$R_{im}^{\left(k\right)}$	The entire feature spaces received from the i^th^ signal measurements of an m^th^ feature space of the k^th^ reference point.
L	# of references point.
$n_{k}$	# of signal measurements received at k^th^ reference point.
$\bar{R}$	The grand/combined mean of the signal measurements of the grid point.
$r_{im}^{\left(k\right)}{|}_{k=0}^{L-1}$	The i^th^ signal measurements of an m^th^ feature space of the k^th^ reference point.
${\bar{r}}_{k}$	The mean signal values of a reference point or a grid point.
$p_{s}$/$p_{T}$	# of sources feature spaces/#Targets feature spaces.
$n_{S}/n_{T}$	# of instances of the sources/Target domains.
$S_{B}$/$S_{w}$	Scatter matrix between/within classes.
$X_{n\times p}$	Feature spaces matrix of dimension n by p.
$Y_{L-1\times p}$	The y label of the L^th^ grid points of the p^th^ feature space.
$W_{p\times L-1}$	The projection matrix of p^th^ features spaces for the corresponding $\left(L-1\right)$ grid points.
$J\left(W\right)$	Determinants of the scatter matrices.
$\rho_{r_{i}r_{j}}$	The correlation between feature spaces.
$z_{im}^{\left(k\right)}$	The i^th^ standardized value of a p^th^ feature space of the k^th^ reference point.
$D_{s}$/$D_{T}$	Source domain/target domain.
$X_{s}^{p}$, $X_{T}^{p}$	The p^th^ features spaces of the source domain data/target domain data.

**Table 2:** Description of the dataset used for adaptive long-term Wi-Fi indoor localization.

Feature	Size (m^2^)	BSs	UEs	#RPs	#BSs	#Features	#Offline Samples	#Testing Samples	Collection Period in Months
RSS	308.4	AP	Smartphone	106	620	620	22,464	3120 per month	25

**Table 3:** Wi-Fi fingerprint-based indoor location estimation of targets utilizing original feature spaces of size 620 with dynamic temporal signal variations (month 1 and 2).

Classifiers	Dataset Collected									
	Month 1 (Only One Training Dataset)					Month 2				
	#MAE (in Meters)									
	#Testing Samples					#Testing Samples				
	1	2	3	4	5	1	2	3	4	5
DT	2.31	2.97	4.41	5.57	2.33	2.30	2.98	4.49	5.73	2.30
KNN	2.26	2.88	4.38	5.71	2.34	2.15	3.11	4.55	5.79	2.15
SVC	2.22	2.87	4.38	5.52	2.28	2.26	3.04	4.50	5.72	2.25
LR	2.21	2.86	4.36	5.62	2.36	2.25	2.99	4.50	5.66	2.25
RF	2.22	3.00	4.49	5.63	2.23	2.26	3.07	4.60	5.76	2.15
GMM	2.23	3.11	4.27	5.67	2.06	2.10	3.10	4.64	5.68	2.03
MLP	2.02	3.49	5.10	6.07	2.04	1.92	3.50	5.14	6.06	1.92
Ada-LT IP	1.98	2.25	3.08	3.97	1.99	1.98	2.55	4.21	5.40	1.99

**Table 4:** Wi-Fi fingerprint-based indoor location estimation of targets utilizing original feature spaces of size 620 with dynamic temporal signal variations (month 3 and 4).

Classifiers	Dataset Collected									
	Month 3 (Only One Training Dataset)					Month 4				
	#MAE (in Meters)									
	#Testing Samples					#Testing Samples				
	1	2	3	4	5	1	2	3	4	5
DT	2.24	3.03	4.45	5.70	2.30	2.28	3.03	4.42	5.69	2.26
KNN	2.19	3.03	4.46	5.76	2.20	2.23	3.09	4.55	5.84	2.26
SVC	2.22	3.02	4.37	5.64	2.30	2.22	2.98	4.52	5.74	2.27
LR	2.23	3.00	4.36	5.60	2.29	2.31	3.01	4.46	5.74	2.33
RF	2.12	3.07	4.49	5.75	2.14	2.15	3.09	4.60	5.81	2.17
GMM	2.13	2.88	4.58	5.62	2.18	2.19	3.17	4.66	5.58	2.08
MLP	1.89	3.53	5.15	6.06	1.92	1.97	2.58	4.13	5.43	1.97
Ada-LT IP	1.97	2.52	4.16	5.41	1.97	1.97	2.54	4.24	5.42	1.97

**Table 5:** Wi-Fi fingerprint-based indoor location estimation of targets utilizing derived feature spaces based on mean signal strength received values (month 1 and 2.).

Classifiers	Dataset Collected									
	Month 1 (Only One Training Dataset)					Month 2				
	#MAE (in Meters)									
	#Testing Samples					#Testing Samples				
	1	2	3	4	5	1	2	3	4	5
DT	2.26	2.98	4.41	5.57	2.36	2.26	3.01	4.43	5.73	2.30
KNN	2.15	2.88	4.38	5.71	2.34	2.15	3.11	4.55	5.79	2.15
SVC	2.26	2.87	4.38	5.52	2.28	2.26	3.04	4.50	5.72	2.25
LR	2.25	2.86	4.36	5.62	2.36	2.25	2.99	4.50	5.66	2.25
RF	2.12	3.00	4.49	5.63	2.27	2.15	3.09	4.52	5.76	2.11
GMM	2.55	2.98	4.27	5.80	2.22	2.34	3.05	4.56	5.68	2.17
MLP	1.92	3.49	5.10	6.07	2.04	1.92	3.50	5.14	6.06	1.92
Ada-LT IP	1.25	1.73	2.61	3.44	1.27	1.26	1.74	2.64	3.50	1.26

**Table 6:** Wi-Fi fingerprint-based indoor location estimation of targets utilizing derived feature spaces based on mean signal strength received values (month 3 and 4).

Classifiers	Dataset Collected									
	Month 3 (Only One Training Dataset/Month)					Month 4				
	#MAE (in Meters)									
	#Testing Samples					#Testing Samples				
	1	2	3	4	5	1	2	3	4	5
DT	2.24	3.03	4.45	5.70	2.30	2.28	3.03	4.42	5.69	2.26
KNN	2.19	3.03	4.46	5.76	2.20	2.23	3.09	4.55	5.84	2.26
SVC	2.22	3.02	4.37	5.64	2.30	2.22	2.98	4.52	5.74	2.27
LR	2.23	3.00	4.36	5.60	2.29	2.31	3.01	4.46	5.74	2.33
RF	2.12	3.07	4.49	5.75	2.14	2.15	3.09	4.60	5.81	2.17
GMM	2.13	2.88	4.58	5.62	2.18	2.19	3.17	4.66	5.58	2.08
MLP	1.89	3.53	5.15	6.06	1.92	1.97	2.58	4.13	5.43	1.97
Ada-LT IP	1.23	1.76	2.66	3.51	1.26	1.24	1.82	2.62	3.48	1.25

**Table 7:** Effect of feature spaces on the variance account of Wi-Fi fingerprint-based indoor location estimation of both training and testing samples datasets (month 1 only).

Dataset Collected								
Month 1								
#Training Samples				#Testing Samples				
#PCA (Explained Variance Ratio in %)								
1	2	3	4	1	2	3	4	5
8 (50%)	13 (50%)	15 (50%)	15 (50%)	19 (50%)	17 (50%)	15 (50%)	15 (50%)	17 (50%)
26 (80%)	39 (80%)	45 (80%)	43 (80%)	52 (80%)	48 (80%)	44 (80%)	47 (80%)	49 (80%)
32 (85%)	46 (85%)	52 (85%)	50 (85%)	61 (85%)	56 (85%)	51 (85%)	55 (85%)	56 (85%)
39 (90%)	54 (90%)	62 (90%)	60 (90%)	72 (90%)	67 (90%)	61 (90%)	65 (90%)	66 (90%)
50 (95%)	67 (95%)	77 (95%)	73 (95%)	72 (95%)	67 (95%)	61 (95%)	65 (95%)	99 (95%)
67 (99%)	87 (99%)	99 (99%)	94 (99%)	110 (99%)	105 (99%)	95 (99%)	103 (99%)	99 (99%)

**Table 8:** Effect of feature spaces on the variance account of Wi-Fi fingerprint-based indoor location estimation of both training and testing sample datasets (different months).

Dataset Collected							
#Training Samples				#Testing Samples			
#PCA (Explained Variance Ratio in %)							
M-1	M-2	M-3	M-4	M-1	M-2	M-3	M-4
8 (50%)	17 (50%)	19 (50%)	16 (50%)	19 (50%)	15 (50%)	19 (50%)	16 (50%)
26 (80%)	51 (80%)	53 (80%)	49 (80%)	52 (80%)	45 (80%)	52 (80%)	45 (80%)
32 (85%)	60 (85%)	62 (85%)	58 (85%)	61 (85%)	54 (85%)	61 (85%)	52 (85%)
39 (90%)	72 (90%)	74 (90%)	70 (90%)	72 (90%)	64 (90%)	73 (90%)	62 (90%)
50 (95%)	88 (95%)	91 (95%)	87 (95%)	72 (95%)	64 (95%)	73 (95%)	62 (95%)
67 (99%)	111 (99%)	114 (99%)	111 (99%)	110 (99%)	100 (99%)	113 (99%)	97 (99%)

**Table 9:** Wi-Fi fingerprint-based indoor location estimation of targets utilizing extracted feature spaces based on principal components (month 2).

Classifiers	Dataset Collected									
	Month 1 (Only One Training Dataset for Each Month)					Month 2				
	#MAE (in Meters)									
	#Testing Sample					#Testing Sample				
	80%	85%	90%	95%	Ref.	80%	85%	90%	95%	Ref.
DT	2.28	2.24	2.24	2.34	2.27	2.32	2.26	2.29	2.32	2.30
KNN	2.15	2.18	2.29	2.37	2.23	2.13	2.06	2.11	2.09	2.20
SVC	2.68	2.79	2.79	2.72	2.52	1.88	1.88	1.88	1.88	2.07
LR	2.20	2.22	2.26	2.27	2.08	2.22	2.27	2.20	2.28	2.20
RF	2.11	2.13	2.11	2.13	2.12	2.09	2.06	2.06	2.05	2.20
GMM	1.92	2.72	1.91	2.04	2.27	1.87	1.94	2.23	2.51	2.01
MLP	2.23	2.23	2.28	2.23	2.21	2.23	2.28	2.23	2.32	2.27
Ada-LT IP	1.68	1.69	1.69	1.98	2.01	1.67	1.68	1.69	1.98	2.00

**Table 10:** Wi-Fi fingerprint-based indoor location estimation of targets utilizing extracted feature spaces based on principal components (month 4).

Classifiers	Dataset Collected									
	Month 3 (Only One Training Dataset for Each Month)					Month 4				
	#MAE (in Meters)									
	#Testing Sample					#Testing Sample				
	80%	85%	90%	95%	Ref.	80%	85%	90%	95%	Ref.
DT	2.29	2.31	2.29	2.26	2.37	2.26	2.19	2.23	2.23	2.36
KNN	2.26	2.28	2.28	2.24	2.21	2.14	2.14	2.18	2.18	2.17
SVC	1.90	1.91	1.90	1.97	2.02	2.63	2.73	2.06	2.06	2.06
LR	2.32	2.29	2.30	2.28	2.28	2.23	2.27	2.31	2.31	2.20
RF	2.03	2.08	2.09	2.05	2.12	2.14	2.07	2.08	2.08	2.07
GMM	1.87	2.34	1.87	2.66	2.31	1.87	1.87	1.87	1.87	2.09
MLP	2.21	2.24	2.26	2.28	2.18	2.19	2.22	2.23	2.23	2.25
Ada-LT IP	1.67	1.68	1.69	1.97	2.00	1.68	1.67	1.69	1.98	2.01

**Table 11:** Wi-Fi fingerprint-based indoor location estimation of targets utilizing extracted feature spaces based on functional discriminants (month 2).

Classifiers	Dataset Collected									
	Month 1 (Only One Training Dataset for Each Month)					Month 2				
	#MAE (in Meters)									
	#Testing Samples					#Testing Samples				
	1	2	3	4	5	1	2	3	4	5
DT	2.27	2.98	4.42	5.73	2.25	2.28	2.96	4.41	5.69	2.26
KNN	2.16	2.98	4.43	5.78	2.19	2.23	3.12	4.54	5.84	2.24
SVC	2.54	3.22	4.19	6.04	2.56	2.27	3.29	4.62	6.00	2.26
LR	2.18	3.01	4.40	5.72	2.27	2.26	3.00	4.43	5.70	2.26
RF	2.10	3.05	4.59	5.81	2.14	2.18	3.07	4.54	5.75	2.14
GMM	2.37	3.10	4.48	5.81	2.13	2.56	3.42	4.38	6.11	2.56
MLP	2.19	3.01	4.41	5.74	2.26	2.27	2.97	4.41	5.71	2.26
Ada-LT IP	1.43	1.91	2.75	3.63	1.53	1.30	1.84	2.66	3.50	1.37

**Table 12:** Wi-Fi fingerprint-based indoor location estimation of targets utilizing extracted feature spaces based on functional discriminants (month 4).

Classifiers	Dataset Collected									
	Month 3 (Only One Training Dataset for Each Month)					Month 4				
	#MAE (in Meters)									
	#Testing Samples					#Testing Samples				
	1	2	3	4	5	1	2	3	4	5
DT	2.23	3.03	4.42	5.72	2.24	2.23	3.01	4.45	5.82	2.24
KNN	2.21	3.08	4.40	5.77	2.27	2.25	3.12	4.54	5.92	2.30
SVC	2.59	3.29	4.19	6.13	2.62	2.68	3.29	4.18	6.13	2.73
LR	2.21	2.99	4.38	5.71	2.25	2.25	3.04	4.50	5.79	2.28
RF	2.15	3.09	4.56	5.78	2.20	2.14	3.06	4.61	5.84	2.15
GMM	2.40	3.04	4.38	5.54	2.36	2.15	2.92	4.30	5.77	2.19
MLP	2.20	3.01	4.36	5.69	2.24	2.24	3.03	4.51	5.81	2.27
Ada-LT IP	1.39	1.81	2.62	3.54	1.40	1.42	1.83	2.63	3.54	1.44

## Data Availability

The dataset used for this study are available upon request to the corresponding author.
